# Suppression of Peierls Instability in a Metal−Halide Porous Framework

**DOI:** 10.1002/anie.7724982

**Published:** 2026-08-23

**Authors:** Ning Zhou, Yangbo Zhang, Ying‐Fan Tan, Qingyun Wan

**Affiliations:** ^1^ Department of Chemistry The Chinese University of Hong Kong Hong Kong SAR China; ^2^ Shanghai‐Hong Kong Joint Laboratory in Chemical Synthesis The Chinese University of Hong Kong Hong Kong SAR China

**Keywords:** MX chains, Peierls distortion, porous frameworks

## Abstract

Peierls distortion is a lattice instability in low‐dimensional materials driven by electron–phonon coupling, which opens an energy gap at the Fermi level and induces a metal–insulator transition. Suppressing this distortion is crucial for stabilizing metallic and superconducting states in low‐dimensional systems, yet achieving such suppression remains challenging without external chemical doping or high‐pressure conditions. Metal–organic framework (MOF) forms a periodic porous structure with well‐defined channels and functional sites. In this work, we harness the intrinsic periodic electrostatic potential of a porous framework to suppress the Peierls instability in one‐dimensional platinum−halide (Pt−X) chains. By integrating Pt−X chains into the ordered nanochannels of the porous material, we achieved suppression of detectable Peierls distortion, as evidenced by the absence of lattice distortion and an approximately four‐order‐of‐magnitude enhancement in its electrical conductivity (from 4.8 × 10^−6^ to 4.3 × 10^−2^ S/cm) compared to the distorted counterparts. This study demonstrates an example where the intrinsic periodic electrostatic potential of a porous framework can be employed to suppress the Peierls distortion, providing new insight for achieving controllable electronic properties in low‐dimensional electronic materials.

## Graphical abstract

Peierls distortion in one‐dimensional Pt‐I chains is suppressed by confinement within the periodic electrostatic environment of a porous Cu–I framework. X‐ray crystallography shows equivalent Pt–I distances without bond alternation, and conductivity of the Pt‐I chains is greatly enhanced. It demonstrates that porous confinement enables effective control of electron‐electron correlations and electron‐phonon couplings in low‐dimensional materials.

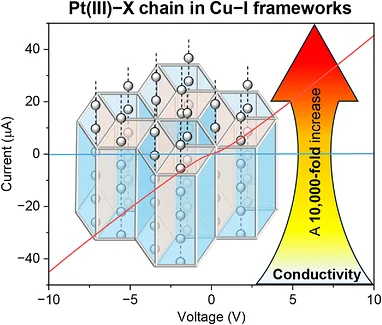

## 1 Introduction

Low‐dimensional materials exhibit a rich variety of electronic phenomena arising from the interplay between lattice degrees of freedom and electron correlations. Among these, the Peierls instability represents a fundamental lattice distortion mechanism in one‐dimensional (1D) systems, where electron‐phonon coupling induces a periodic lattice modulation that opens an electronic band gap at the Fermi level, driving a metal‐insulator transition (Figure 1a−d) [1, 2]. This mechanism underlies a wide range of phenomena in low dimensional materials, including the formation of charge density waves (CDWs) and temperature dependent metal‐insulator transitions [1, 2, 3, 4, 5]. Consequently, suppressing the Peierls distortion has become a key strategy for stabilizing low‐dimensional metals and accessing exotic electronic ground states such as superconductivity [3, 4].

**FIGURE 1:**
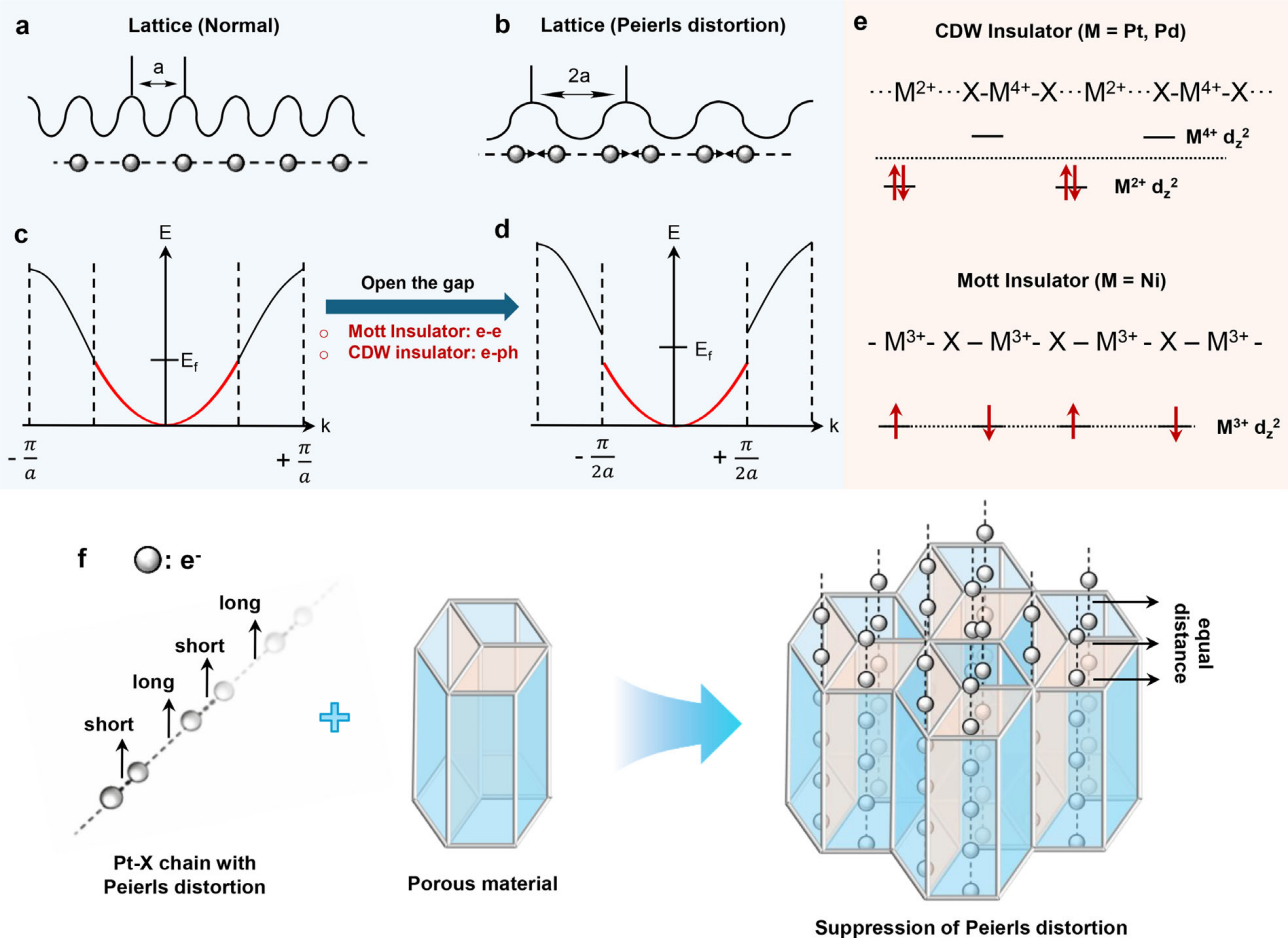
(a, b), Schematic drawing showing a periodic lattice Peierls distortion in the CDW insulator. (c,d), Energy band gaps before and after considering strong electron‐electron (e‐e) correlations in a Mott insulator or strong electron‐phonon (e‐ph) couplings in a CDW insulator for the one‐dimensional system. After considering the e‐e or e‐ph interaction, the on‐site Coulomb repulsion or lattice distortion results in an energy gap at the Fermi level E_f_. (e), Electronic structures of MX chain for a CDW insulator when M = Pt or Pd, and Mott insulator when M = Ni. (f), Schematic drawing showing the encapsulation of Pt−X chains within a porous material, leading to the suppression of the Peierls distortion.

Conventional approaches to suppress Peierls distortions typically rely on external chemical doping, high‐pressure conditions, or lattice strain, all of which alter the electronic structure or alter the balance of electron‐phonon coupling [4, 6, 7]. However, each of these strategies has limitations because they can introduce disorder, require complex processing, or change other material properties in undesirable ways. Metal−organic frameworks (MOFs) and porous coordination polymers (PCPs) have emerged as a versatile class of crystalline materials characterized by their periodic structures, which feature ordered nanochannels and tunable electrostatic environments [8, 9, 10]. The intrinsic periodic electrostatic potential inherent to the MOF architecture presents a unique opportunity to modulate the electronic behavior of guest species confined within its pores, potentially providing a new pathway to control lattice instabilities.

In this work, we exploit the periodic electrostatic potential intrinsic to a porous architecture to stabilize a one‐dimensional Pt−X chains against Peierls dimerization. Recent work has demonstrated the use of MOFs as templates to direct the confined growth of metal‐halide units (e.g., NiBr_2_, NiCl_2_, CoCl_2_, FeCl_2_, MnBr_2_, PbI_2_, PbBr_2_), resulting in a family of single‐crystalline porous superlattice structures with precise atomic coordinates [11, 12, 13, 14, 15, 16, 17, 18, 19]. Here, we realize this concept using a porous coordination host composed of three‐dimensional Cu−I inorganic assemblies with ordered 1D nanochannels, into which one‐dimensional Pt−I chains are integrated (Figures 1 and 2). Halide‐bridged platinum chains (Pt−X chains) are notable for their pronounced Peierls instability and robust CDW orders (Figure 1e) [20, 21, 22, 23, 24, 25, 26, 27, 28, 29, 30, 31, 32, 33, 34, 35]. Traditionally, the ground state in these systems is described as a mixed‐valence charge order alternating between Pt(II) and Pt(IV) sites accompanied by strong lattice distortion. In this work, these Pt−X (X = I) chains are confined within ordered nanochannels in a porous framework, and we compare their structural and transport properties to those of isolated or distorted Pt−X (X = Br, I) chains. Structural characterization reveals the absence of the characteristic lattice distortion associated with the Peierls state and indicates an averaged Pt(III) oxidation state in Pt−I@pores structures. Electrical measurements show an increase in conductivity of roughly four orders of magnitude, demonstrating that suppression of the Peierls gap restores high conductivity by confining inside the nanochannels of a porous structure.

**FIGURE 2:**
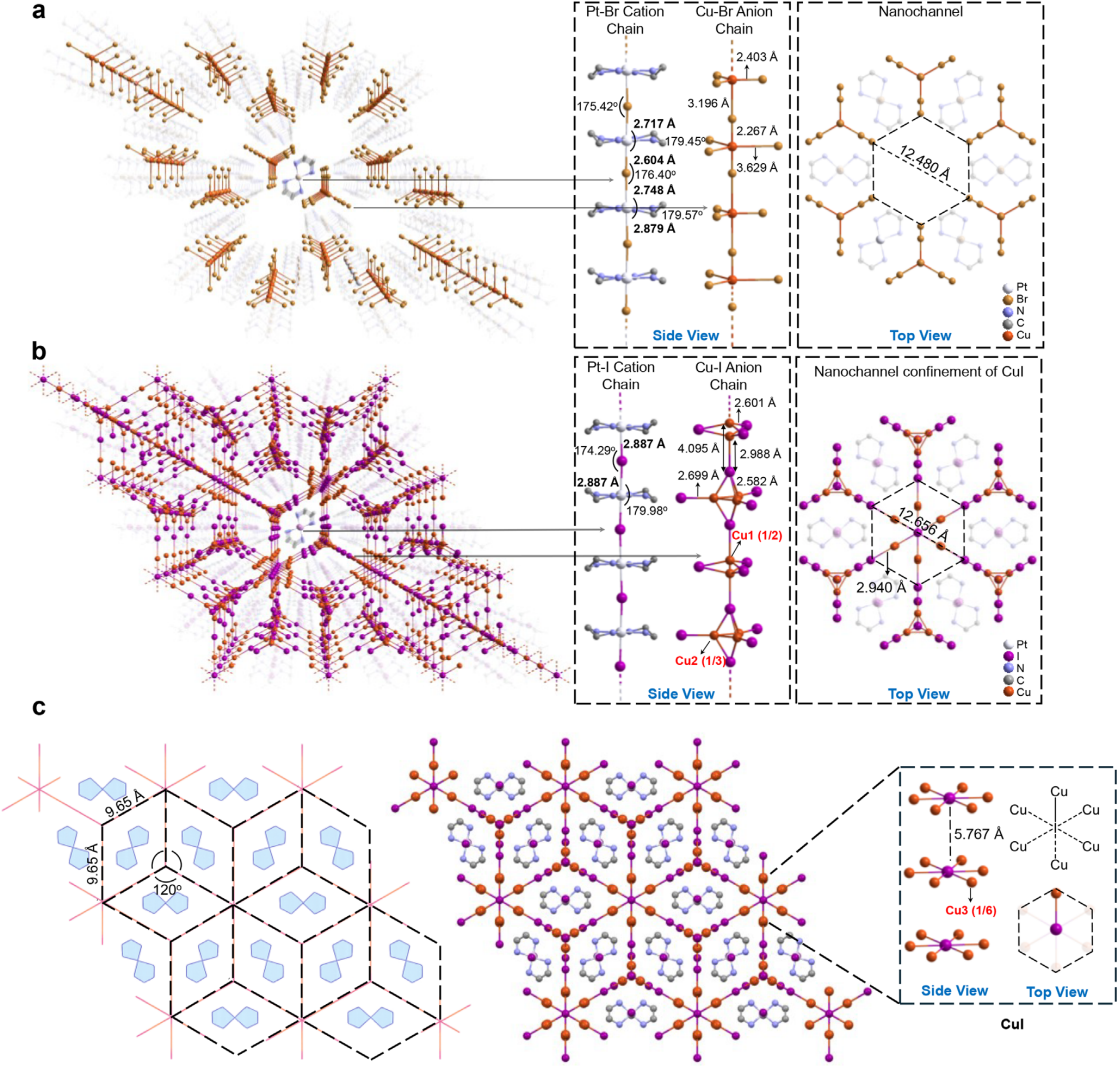
The crystal structure of (a), compound **1** and (b), compound **2** at 293 K. (c), Top view of the crystal structure of compound **2**. Hydrogen atoms were omitted for clarity. White atom: Pt; purple atom: I; grey atom: C; blue atom: N; yellow atom: Br; orange atom: Cu.

These results establish that the intrinsic periodic electrostatic potential in porous materials can serve as a noninvasive and generalizable design principle to prevent lattice instabilities in low‐dimensional conductors. Notably, suppression of Peierls distortion of confined low‐dimensional materials has been observed reported in various porous hosts, including carbon nanotubes and porous materials such as zeolites [36, 37, 38]. We believe this strategy may open new pathways for designing porous electronic materials with high mobility and tunable quantum phases.

## 2 Result and Discussion

Two compounds, {[Pt(en)_2_][PtBr_2_(en)_2_]}_3_[CuBr_4_]_4_ (en = ethylenediamine, compound **1**) and [PtI(en)_2_]_3_(CuI_4_)_2_CuI (compound **2**), were prepared and characterized in this work. Compound **1** has been reported previously, compound **2** is new and was synthesized using a modified literature procedure (see Supporting Information) [39]. These two compounds both feature alternating anionic CuX and cationic Pt−X chain or assembly structures (X = Br or I, Figure 2). The chemical and physical properties of low‐dimensional MX chain systems (M = Ni/Pd/Pt, X = Cl/Br/I) have attracted significant research interest due to their intrinsically unique physical properties (Figure 1) [31, 32, 33]. In the ground state of Ni‐based compounds, no charge disproportionation or lattice distortion is observed (Figure 1e), featuring a Mott insulating state. While in the Pd/Pt‐based systems, X atoms are typically displaced from the midpoint between adjacent Pt atoms (Peierls distortion), inducing charge disproportionation (mixed valency) on the metal (M) atoms (Figure 1b,d). This charge disproportionation drives the formation of a CDW state, where the ground state consists of mixed‐valent M(II)/M(IV) chains [20]. The CDW arises from an alternating arrangement of electron pairs in the Pt‐5d_z_^2^ orbitals (Figure 1e). A classical example is the complex of [Pt(en)_2_][PtI_2_(en)_2_](ClO_4_)_4_ (reference compound used in this work) which exemplifies this structural and electronic configuration [20, 40, 41]. In this work, we prepared the Pt−X chain compounds where the Cu−X chains/assembles behave as the counter‐anion in compounds **1** and **2**.

The lattice distortion induced by the halide X atom serves as an important indicator of the Peierls instability and the CDW state (Figure 1b,d). Comparison of the single‐crystal structures reveals obvious lattice distortion of the Pt−Br bond, indicative of the Peierls instability in compound **1**, whereas such distortion is absent in the Pt−I bond of compound **2** (Figure 2). The crystal structures of compounds **1** and **2** are shown in Figure 2a,b. Compound **1**, {[Pt(en)_2_][PtBr_2_(en)_2_]}_3_[CuBr_4_]_4_, exhibits a double‐chain structure in which the [CuBr_4_] units form an anionic chain and the [Pt(en)_2_][PtBr_2_(en)_2_] units form a cationic chain. In compound **2**, [PtI(en)_2_]_3_(CuI_4_)_2_CuI, the anionic [CuI_4_] chains together with the integrated CuI units constitute porous framework structure that hosts the cationic Pt−I chain within its quadrilateral nanochannels.

As shown in Figure 2a, the planar [Pt(en)_2_] units in compound **1** are bridged by bromide ions, forming a slightly bent Pt−Br chain with a Pt−Br−Pt angle of 175.42° and 176.40°. Alternating long and short Pt−Br distances (2.717, 2.604, 2.748, and 2.879 Å) are observed along the Pt−Br infinite chain. The Pt atoms with longer Pt−Br distances (2.748 and 2.879 Å) are assigned as Pt(II), while those with shorter Pt−Br distances (2.717 and 2.604 Å) are assigned as Pt(IV). This alternation of short and long Pt−Br distances suggest the presence of the Peierls instability and CDW order [20, 21, 22, 23, 24, 25, 26, 27, 28, 29, 30, 31, 32, 33, 34, 35]. In parallel, the Cu(I) ions adopt a quasi‐trigonal‐bipyramidal geometry and are bridged by bromide ions to form a one‐dimensional anionic chain. This Cu−Br chain displays alternating long and short distances of 3.196 and 2.267 Å, respectively.

As shown in Figure 2b, the Cu−I chain in compound **2** adopts a structure analogous to that of the Cu−Br chain in compound **1**. Along the main chain direction, the disordered Cu1−I distances are 2.988 and 2.601 Å, and the Cu2−I distance is 2.582 Å. Unlike compound **1**, the Cu−I chains are further linked by [CuI] units, resulting in the formation of a porous framework structure. For the [CuI] unit, the Cu3 site is occupied with 1/6 occupancy, while the central I site is fully occupied. The resolved Pt−I chain is located within the nanosized channels of the (CuI_4_)_2_CuI assembly, revealing a slightly bent Pt−I chain with a Pt−I−Pt angle of 174.29° and Pt···Pt and Pt−I distances of 5.767 and 2.887 Å, respectively. This slightly bent Pt−I−Pt angle (174.29°), a small deviation from linearity, may influence orbital overlap and band dispersion, potentially affecting the electronic structure and transport properties. Notably, each Pt─I bond distance is equivalent at 2.887 Å. Based on this equal Pt─I bond length, an averaged Pt(III) oxidation state is assigned for compound **2**.

These structural comparisons highlight the significant influence of the assembled (CuI_4_)_2_CuI framework structures on the charge order within the Pt−X chains. By confining the Pt−I chains within this framework structure, lattice distortion and CDW order are suppressed in compound **2**—a stark contrast to compound **1**. We have also resolved the crystal structures of compound **2** at low temperature (100 K). No detectable Peierls‐type bond alternation was observed for compound **2** in the crystallographic structure at 100 K, similar to the equal Pt–I distances observed at 293 K (Figure S8). This demonstrates that the suppression of the Peierls distortion persists over a wider temperature range. Compound **2** was further compared with the reference compound **[Pt(en)_2_][PtI_2_(en)_2_](ClO_4_)_4_**. This reference compound represents an isolated, non‐confined Pt−I chain system without a porous framework [40, 41]. By comparing this reference compound with compound **2**, we can more rigorously isolate the effect of porous confinement on the Peierls distortion. As shown in Figure S2, both the reference compound and compound **2** feature Pt−I chains. The key difference lies in the counter‐anion: ClO_4_^−^ for the reference compound versus the Cu−I framework for compound **2**. The Cu−I framework constitutes the porous host that confines the Pt−I chains within its pores. This confinement leads to a dramatic change in the Pt−I distance pattern: the reference compound displays alternating long and short Pt−I distances (3.115 and 2.705 Å), indicative of significant Peierls distortion, whereas compound **2** exhibits equal Pt−I distances (2.887 Å), indicating suppression of the Peierls distortion. This direct comparison rigorously demonstrates that porous confinement effectively suppresses the Peierls distortion and leads to equal Pt─I bond distances in compound **2**.

Thermogravimetric analysis was performed on compounds **1** and **2** to test their thermal stability. As shown in Figure S3, the thermogravimetric analysis (TGA) data of compound **1** reveals a weight loss of 3.5% upon heating from room temperature to 120°C. This weight loss is attributed to the removal of solvent molecules (H_2_O) trapped within the lattice voids during heating, which is consistent with the elemental analysis results of this compound. In contrast, compound **2** exhibits negligible weight change over the same temperature range. Above 260°C, both compounds show a sharp decrease in weight, indicating the onset of structural decomposition. Upon heating to 800°C, the residual masses correspond essentially to Cu and Pt only.

Stabilizing the Pt(III) valence state in Pt−X chain systems has long been a great challenge [42]. The Mott‐Hubbard model has been widely used to elucidate the underlying reason for this challenge, where a large electron transfer integral (*t*) and a small on‐site Coulomb repulsion (*U*) typically compete with one another in the Pt‐based MX chain systems [43, 44, 45]. As a result, a mixed‐valence Pt(II)/Pt(IV) state accompanied by lattice distortion has been widely observed in Pt−X chains [33]. In contrast, the Pt−I chain encapsulated in the Cu−I framework structure reported in this work exhibits no obvious lattice distortion revealed by the equal Pt−I bond length (Figure 2). This unusual observation motivates further spectroscopic investigation to elucidate the detailed electronic structure of this compound. In the following sections, we employ various spectroscopic methods, including Raman spectroscopy, X‐ray photoelectron spectroscopy (XPS), optical and transport measurements, to further probe the electronic structures of compounds **1** and **2** and elucidate their differences.

Raman spectra were measured to obtain information about the charge orders in compound **1** and **2**. In Figure 3a, the strong band at 174 cm^−1^ is attributed to the Pt−Br−Pt stretching mode, υ_1_(Pt−Br−Pt), for compound **1** [39]. An overtone progression of υ_1_(Pt−Br−Pt) up to fifth order (348, 522, 696, 870 cm^−1^) is observed in this Raman spectra. The presence of these overtones indicates strong electron‐phonon coupling and anharmonicity in the potential energy surface of the Pt−Br−Pt stretching vibrational modes. It is important to note that, for MX chain systems, only those with lattice distortions exhibit this Raman‐active M−X stretching mode, because this mode leads to a change in polarizability only when unequal Pt−X bonds exist in the chain. The observed strong Pt−Br−Pt stretching mode and its overtones indicate the presence of lattice distortion and unequal Pt−Br bond lengths, which is consistent with the crystal structure shown in Figure 2a. A weak Raman peak at ∼578 cm^−1^ is assigned to the υ_2_(Pt−N) stretching mode in compound **1**, in agreement with previous reported work [39]. A weak peak at 752 cm^−1^ is also present in Figure 3a and is assigned to a combination band of the υ_1_ + υ_2_ modes.

**FIGURE 3:**
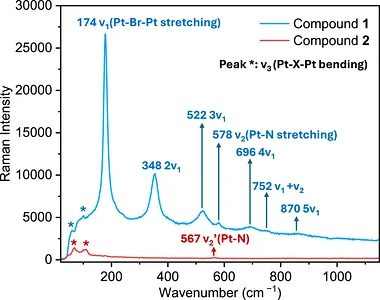
Raman spectra of the single crystal sample for compound **1** and compound **2** at room temperature (*λ*_exc_ = 532 nm).

In the Raman spectra measured for compound **2**, neither the Raman peak of the Pt−I stretching mode nor its overtones were observed in Figure 3a. Similarly, the Pt−N stretching mode for compound **2** is observed at 567 cm^−1^. The absence of the Pt−I stretching vibrational modes in Figure 3a suggests the disappearance of CDW order in the Pt−I chain, which is consistent with the observed crystal structures showing no lattice distortion in Figure 2. Notably, two low‐energy Raman peaks were observed for both compound **1** and compound **2**, located at 64 and 100 cm^−1^ for compound **1**, and at 69 and 110 cm^−1^ for compound **2**. These low‐energy peaks were tentatively assigned to the Pt−X−Pt bending mode, as the Pt−X−Pt chain is not strictly straight; slight bend angles of and 174.29°−176.40° were observed in the crystal structures of compound **1** and compound **2**, respectively. These Raman peaks for compounds **1** and **2** are summarized in Table 1 for comparison.

**TABLE 1:** Raman peaks (cm^−1^) and assignments of bands observed in the Raman spectrum of compound **1** and compound **2**.

Raman peaks (cm^−1^)	Compound 1	Raman peaks (cm^−1^)	Compound 2
64	υ_3_ (Pt−Br−Pt bending)	69	υ_3_' (Pt−I−Pt bending)
100	υ_3_ (Pt−Br−Pt bending)	110	υ_3_' (Pt−I−Pt bending)
174	υ_1_ (Pt−Br−Pt stretching)	567	υ_2_' (Pt−N)
348	2υ_1_		
522	3υ_1_		
578	υ_2_ (Pt−N)		
696	4υ_1_		
752	υ_1_ + υ_2_		
870	5υ_1_		

To further assign the oxidation states of the Pt centers in compounds **1** and **2**, we performed XPS on the compounds as well as on their starting materials/building blocks, including [Pt(II)(en)_2_]Cl_2_ and [Pt(IV)I_2_(en)_2_]Cl_2_. Figure 4a–d show high‑resolution Pt 4f spectra (4f_5/2_ and 4f_7/2_). As summarized in Table 2 and illustrated in Figure 4a, [Pt(II)(en)_2_]Cl_2_ exhibits Pt 4f peaks at 72.3 eV (Pt(II) 4f_7/2_) and 75.6 eV (Pt(II) 4f_5/2_) [46]. By contrast, [Pt(IV)I_2_(en)_2_]Cl_2_ shows shifted, higher‑energy peaks at 74.0 eV (Pt(IV) 4f_7/2_) and 77.3 eV (Pt(IV) 4f_5/2_), consistent with the expected increase in binding energy for the higher oxidation state [47]. Notably, the Pt(IV) compound is well known to display partial surface reduction to Pt(II) under x‐ray irradiation, evidenced by emergent Pt(II) 4f features in Figure 4b [47]. The XPS spectrum of compound **1** shows four peaks at 72.3, 73.9, 75.5, and 77.2 eV. These peak positions closely match those of the [Pt(IV)I_2_(en)_2_]Cl_2_ compound (Figure 4c), but their relative intensities differ. In compound **1**, the peaks corresponding to Pt(II) species exhibit higher intensity compared to those in [Pt(IV)I_2_(en)_2_]Cl_2_. Based on the XPS data, a mixed‐valence Pt(II, IV) oxidation state is assigned for compound **1**, which is consistent with the x‐ray crystal and Raman spectroscopic results.

**FIGURE 4:**
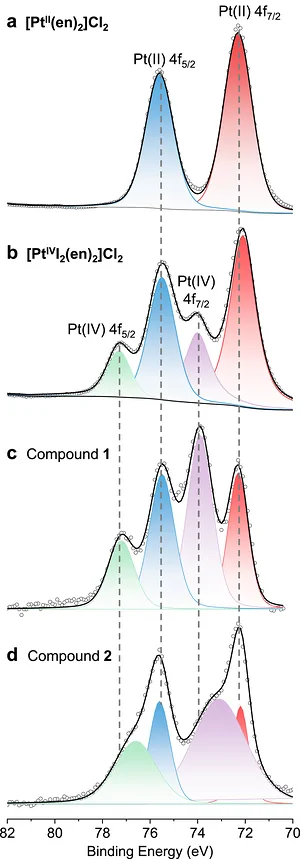
XPS spectra of the Pt 4f core levels for (a), [Pt(en)_2_]Cl_2_, (b), [PtI_2_(en)_2_]Cl_2_, (c), compound **1**, and (d), compound **2**. Black open circles: measured spectrum, black line: fitting curve, blue, red, purple, and green curves: components of the fitting curve.

**TABLE 2:** XPS results. The binding energies (eV) for Pt 4f core levels of [Pt(en)_2_]Cl_2_, [PtI_2_(en)_2_]Cl_2_, compounds **1** and **2**.

Compound	Pt 4f_5/2_	Pt 4f_7/2_
**[Pt(en)_2_]Cl_2_**	75.6	72.3
**[PtI_2_(en)_2_]Cl_2_**	75.5, 77.3	72.1, 74.0
**Compound 1**	75.5, 77.2	72.3, 73.9
**Compound 2**	75.6, 76.6	72.2, 73.1

The XPS spectra of compound **2** are shown in Figure 4d and are further compared with those in Figure 4a–c. Two XPS bands at 72.2 and 75.6 eV were observed and fitted, which are assigned to the Pt(II) 4f_7/2_ and Pt(II) 4f_5/2_ bands, respectively. Two additional peaks appear at 73.1 and 76.6 eV. The 73.1 eV peak lies between the Pt(II) and Pt(IV) positions for the Pt 4f_7/2_ band, and the 76.6 eV peak lies between the Pt(II) and Pt(IV) positions for the Pt 4f_5/2_ band. These intermediate binding energies indicate the presence of Pt(III) atoms in compound **2**. The lower‐binding‐energy doublet at 72.2/75.6 eV corresponds to Pt(II)‐like surface species arising from x‐ray‐induced reduction during measurement, and is observed exclusively upon x‐ray irradiation [47]. In contrast to Figure 4b,c, no distinct Pt(IV) peaks are observed for compound **2** in Figure 4d. These XPS results indicate the presence of an averaged Pt(III) oxidation state rather than a mixed‐valence Pt(II, IV) state in the chain structures of compound **2**, which is consistent with its x‐ray crystal structure (showing no lattice distortion) and Raman spectrum (showing no Pt−I stretching active mode).

The assignment of a Pt(III) oxidation state along the Pt−I chain in compound **2** suggests each Pt center may carry an unpaired electron in its 5d_z_^2^ orbital. To probe for isolated spins, we measured the magnetic susceptibility of compound **1** and **2**. Both samples exhibit small and negative magnetic susceptibility, indicating their diamagnetic behaviors without significant unpaired electrons (Figure S4b). This diamagnetism mirrors that reported for the discrete molecule [Pt(III)_2_I_3_(en)_4_]I_3_ molecule [42]. Referring to the case of compound [Pt(III)_2_I_3_(en)_4_]I_3_, we propose that the strong antiferromagnetic coupling between neighboring Pt(III) centers in compound **1**, mediated by orbital overlap interactions through the Pt‐5d_z_^2^ and I‐5p_z_ orbitals, leading to the observed diamagnetic behavior [42].

Two‐probe DC electrical conductivity measurements were performed on the single crystals of compounds **1** and **2** at room temperature (Figure 5a). The results show a large difference in conductivity: approximately 4.83 × 10^−6^ S cm^−1^ for compound **1** and 4.25 × 10^−2^ S cm^−1^ for compound **2** (Figure 5a). Figure S5 shows I−V curves for three independent samples of compounds **1** and **2**. The consistent resistance validates reproducibility and eliminates contact artifacts. This four order of magnitude difference indicates that encapsulating Pt−I chains within the Cu−I framework structure significantly enhances the conductivity of compound **2**. The conductivity of the reference compound [Pt(en)_2_][Pt(en)_2_I_2_](ClO_4_)_4_ (Pt−I chain with Peierls instability) was further compared with that of compound **2** (Pt‐I chain without Peierls instability) here. The room‐temperature single‐crystal conductivity is 1.6×10^−8^ S cm^−1^ for the reference compound [41].

**FIGURE 5:**
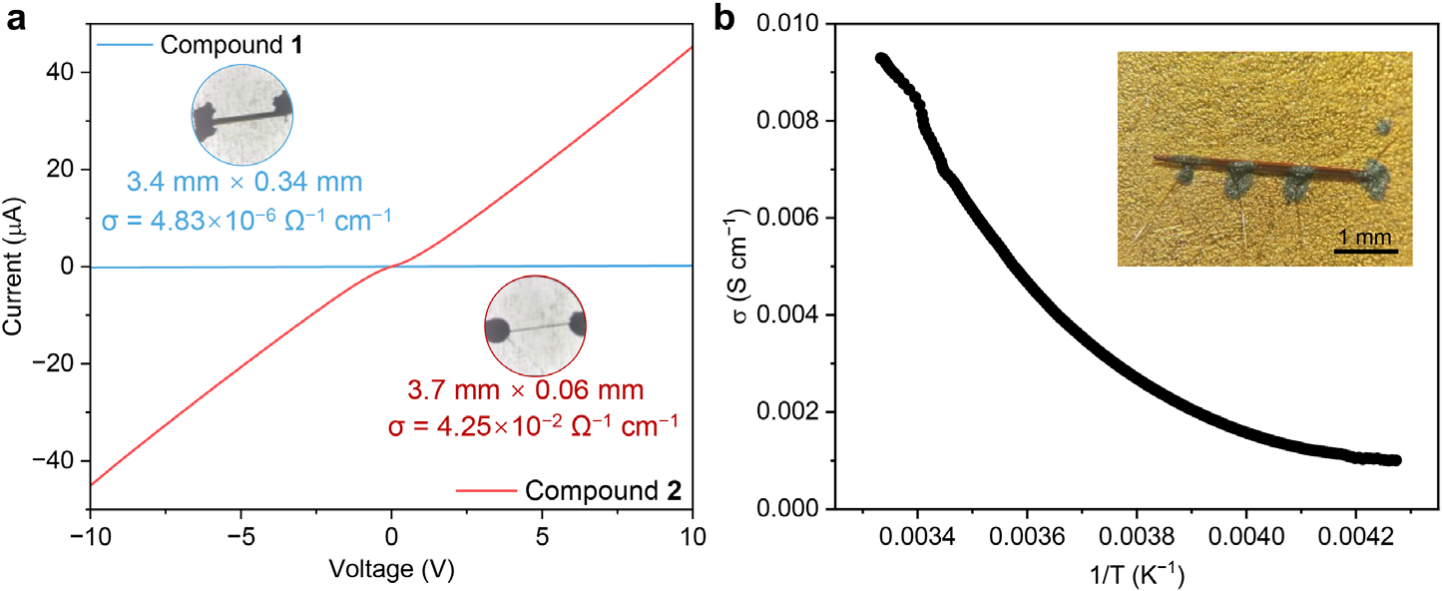
(a), Two‐probe electrical measurements for the single crystal of compounds **1** and **2** at room temperature. (b), Temperature dependence of electrical conductivity σ (S·cm^−1^) for a single crystal of compound **2**. The inset in figure a and b shows the device structure.

This enhancement of conductivity in compound **2**, compared to compound **1** and/or the reference compound, has been attributed to two possible factors: (1) A better orbital overlap between Pt‐5d_z_^2^ and I‐5p_z_ orbitals compared to that between Pt‐5d_z_^2^ and Br‐4p_z_ orbitals. The improved orbital overlap implies stronger charge transfer ability (a larger transfer integral *t*), which promotes higher mobility in compound **2** than in compound **1**. (2) The construction of a periodic electrostatic potential field using an anionic porous framework. Higher dimensional (2D or 3D) systems are generally less prone to Peierls phase transitions than one‐dimensional (1D) systems [1, 2]. In 1D, the Fermi surface is perfectly nested, which strongly favours the Peierls instability and leads to a CDW state. In contrast, the higher dimensionality suppresses perfect nesting and reduces the electron–phonon coupling effects that drive the transition, making the Peierls distortion less favorable. We consider that the electrostatic interaction between the anionic CuI assemblies and cationic Pt−I chain suppresses the Peierls transition in an extended framework structure. Based on the Mott‐Hubbard model, we expect that the presence of this higher dimensional electrostatic potential field will modify the onsite Coulomb repulsion *U* between electrons on adjacent Pt orbitals. This change of *U* may affect the degree of electron localization and thereby, influencing the charge transport ability.

Single‐crystal temperature‐dependent resistivity measurements were further performed on compound **2** using the four‐probe method along the long axis of the crystal. As shown in Figure 5b, the σ vs. T^−1^ relationship exhibits semiconducting behavior, with resistivity decreasing as temperature increases. The plot of ln(σ) versus T^−1^ is linear (Figure S6). Fitting the data with a linear function yields an activation energy (ΔE) of 228 meV at ambient pressure, which corresponds to the energy difference between the transport level and the Fermi level of compound **2**. This semiconducting behavior indicates the opening of an energy gap in its band structure. No lattice distortion was observed in compound **2**, suggesting the absence of electron‐phonon coupling. Instead, electron‐electron repulsion (Figure 1c) is proposed to be responsible for the opening of the energy gap in compound **2**.

To further evaluate the electrostatic potential generated by the CuI porous framework and the electronic configuration of compound **2**, we performed DFT calculations. As shown in Figure S7a, the Cu−I assembly generates a negative electrostatic potential that encapsulates the Pt−I chain. As previously reported for MX−chain systems, the position of the counter‐anions can significantly tune the electron‐pairing site [48]. When the counter‐anion resides in the Pt plane, the Coulombic repulsion from the counter‐anions suppresses electron pairing at individual Pt atoms while promoting pairing between two adjacent Pt atoms (delocalized Pt−I−Pt site pairing). In this scenario, the lattice displacement originates from the Pt atoms [48]. Conversely, when the counter‐anion lies in the I plane, it suppresses the delocalized Pt−I−Pt pairing and favors pairing at a localized single Pt site, with the lattice displacement now driven by the I atoms. In the case of compound **2**, the counter‐anions (I^−^ and/or CuI assemblies) are present in both the Pt and I planes (Figure S7a). This arrangement is expected to generate substantial Coulombic repulsion at both sites and thereby suppress electron pairing at either the delocalized Pt−I−Pt or localized Pt site, resulting in equal Pt−I distances and the absence of lattice distortion. We also performed molecular orbital (MO) calculations for compound **2**. As depicted in Figure S7b, the MO analysis reveals a delocalized Pt−I bond characterized by strong orbital overlap between the Pt‐5d_z_^2^ and I‐5p_z_ orbitals. This overlap gives rise to a strong antiferromagnetic interaction between neighboring Pt atoms, and consequently, to diamagnetism.

## 3 Conclusions

In this work, we have demonstrated that the intrinsic periodic electrostatic potential of a porous metal−halide framework can be harnessed to suppress the Peierls instability in one‐dimensional Pt−X chains. By confining Pt−I chains within the ordered nanochannels of a porous Cu−I assembly, we obtained a material (compound **2**) with equal Pt−I bond lengths, a uniform Pt(III) oxidation state, and no detectable CDW order. This is in sharp contrast to the distorted, mixed‐valence Pt(II)/Pt(IV) state observed in the analogous Pt−Br chain compound **1**.

The suppression of Peierls distortion leads to a nearly four‐order‐of‐magnitude enhancement in electrical conductivity compared to the distorted counterpart. Our findings shed light on the control of charge orders in low‐dimensional systems: using the built‐in electrostatic potential of a porous framework to modulate electron‐phonon and electron‐electron interactions. This approach avoids the disorder and complexity associated with chemical doping or mechanical strain and opens a pathway toward intrinsically conductive porous electronic materials with potential applications in low‐dimensional electronics.

## Author Contributions

**Ning Zhou**: investigation, data curation, writing – review and editing, formal analysis, methodology. **Yangbo Zhang**: investigation, formal analysis. **Ying–Fan Tan**: investigation, formal analysis. **Qingyun Wan**: conceptualization, writing – original draft, funding acquisition, data curation, supervision, writing – review and editing, methodology, investigation.

## Conflicts of Interest

The authors declare no conflicts of interest.

## Supporting information



The authors have cited additional references within the Supporting Information [39−41, 49−59].**Supporting File**: anie74359‐sup‐0001‐SuppMat.pdf.

## Data Availability

The data that support the findings of this study are available from the corresponding author upon reasonable request.

## References

[1] G. Grüner , “The Dynamics of Charge‐Density Waves,” Reviews of Modern Physics 60 (1988): 1129, 10.1103/RevModPhys.60.1129.

[2] R. E. Peierls , Quantum Theory of Solids (Oxford University Press, 1955).

[3] S. Gerber , H. Jang , H. Nojiri , et al., “Three‐Dimensional Charge Density Wave Order in YBa_2_Cu_3_O_6.67_ at High Magnetic Fields,” Science 350 (2015): 949–952, 10.1126/science.aac6257. 26541608

[4] G. Campi , A. Bianconi , N. Poccia , et al., “Inhomogeneity of Charge‐Density‐Wave Order and Quenched Disorder in a High‐Tc Superconductor,” Nature 525 (2015): 359–362, 10.1038/nature14987. 26381983

[5] X. Teng , L. Chen , F. Ye , et al., “Discovery of Charge Density Wave in a Kagome Lattice Antiferromagnet,” Nature 609 (2022): 490–495, 10.1038/s41586-022-05034-z. 36104552

[6] T. Fu , K. Bu , X. Sun , et al., “Manipulating Peierls Distortion in Van Der Waals NbOX_2_ Maximizes Second‐Harmonic Generation,” Journal of the American Chemical Society 145 (2023): 16828–16834, 10.1021/jacs.3c04971. 37467160

[7] T. Fujita , Y. Chen , Y. Kono , et al., “Pressure‐Induced Reversal of Peierls‐Like Distortions Elicits the Polyamorphic Transition in GeTe and GeSe,” Nature Communications 14 (2023): 7851, 10.1038/s41467-023-43457-y. PMC10703813 38062025

[8] O. M. Yaghi , G. Li , and H. Li , “Selective Binding and Removal of Guests in a Microporous Metal–Organic Framework,” Nature 378 (1995): 703–706, 10.1038/378703a0.

[9] B. F. Hoskins and R. Robson , “Infinite Polymeric Frameworks Consisting of Three Dimensionally Linked Rod‐Like Segments,” Journal of the American Chemical Society 111 (1989): 5962–5964, 10.1021/ja00197a079.

[10] M. Fujita , Y. J. Kwon , S. Washizu , and K. Ogura , “Preparation, Clathration Ability, and Catalysis of a Two‐Dimensional Square Network Material Composed of Cadmium (II) and 4,4'‐bipyridine,” Journal of the American Chemical Society 116 (1994): 1151–1152, 10.1021/ja00082a055.

[11] M. I. Gonzalez , A. B. Turkiewicz , L. E. Darago , et al., “Confinement of Atomically Defined Metal Halide Sheets in a Metal–Organic Framework,” Nature 577 (2020): 64–68, 10.1038/s41586-019-1776-0. 31739311

[12] W. Zhang , H. Jiang , Y. Liu , et al., “Metal‐Halide Porous Framework Superlattices,” Nature 638 (2025): 418–424, 10.1038/s41586-024-08447-0. 39910312

[13] A. Turkiewicz , W. Tomlinson , M. I. Gonzalez , J. P. Hooper , and J. R. Long , “Templated Growth of a Spin‐Frustrated Cluster Fragment of MnBr_2_ in a Metal–Organic Framework,” Inorganic Chemistry 60 (2021): 16103–16110, 10.1021/acs.inorgchem.1c01345. 34632759

[14] X.‐N. Wang , P. Zhang , A. Kirchon , et al., “Crystallographic Visualization of Postsynthetic Nickel Clusters Into Metal–Organic Framework,” Journal of the American Chemical Society 141 (2019): 13654–13663, 10.1021/jacs.9b06711. 31398288

[15] W. M. Bloch , A. Burgun , C. J. Coghlan , et al., “Capturing Snapshots of Post‐Synthetic Metallation Chemistry in Metal–Organic Frameworks,” Nature Chemistry 6 (2014): 906–912, 10.1038/nchem.2045. 25242486

[16] Y. Inokuma , T. Arai , and M. Fujita , “Networked Molecular Cages as Crystalline Sponges for Fullerenes and Other Guests,” Nature Chemistry 2 (2010): 780–783, 10.1038/nchem.742. 20729900

[17] H. Xie , M. A. Khoshooei , M. Mandal , et al., “Introducing Metal–Sulfur Active Sites in Metal–Organic Frameworks via Post‐Synthetic Modification for Hydrogenation Catalysis,” Nature Chemistry 17 (2025): 1514–1523, 10.1038/s41557-025-01876-y. 40707779

[18] C. R. Kim , T. Uemura , and S. Kitagawa , “Inorganic Nanoparticles in Porous Coordination Polymers,” Chemical Society Reviews 45 (2016): 3828–3845, 10.1039/C5CS00940E. 27051891

[19] Z.‐F. Wu , C. Wang , X. Liu , et al., “Confinement of 1D Chain and 2D Layered CuI Modules in K‐INA‐R Frameworks via Coordination Assembly: Structure Regulation and Semiconductivity Tuning,” Journal of the American Chemical Society 145 (2023): 19293–19302, 10.1021/jacs.3c05095. 37616202

[20] J. T. Gammel , A. Saxena , I. Batistić , A. R. Bishop , and S. R. Phillpot , “Two‐Band Model for Halogen‐Bridged Mixed‐Valence Transition‐Metal Complexes. I. Ground State and Excitation Spectrum,” Physical Review B 45 (1992): 6408–6434, 10.1103/PhysRevB.45.6408. 10000405

[21] S. Takaishi , M. Takamura , T. Kajiwara , et al., “Charge‐Density‐Wave to Mott−Hubbard Phase Transition in Quasi‐One‐Dimensional Bromo‐Bridged Pd Compounds,” Journal of the American Chemical Society 130 (2008): 12080–12084, 10.1021/ja8032026. 18707181

[22] H. Matsuzaki , H. Kishida , H. Okamoto , et al., “Vapochromic Behavior Accompanied by Phase Transition Between Charge‐Polarization and Charge‐Density‐Wave States in a Quasi‐One‐Dimensional Iodine‐Bridged Dinuclear Platinum Compound,” Angewandte Chemie International Edition 44 (2005): 3240–3243, 10.1002/anie.200500091. 15825215

[23] M. Wakizaka , S. Kumagai , H. Wu , et al., “Macro‐ and Atomic‐Scale Observations of a One‐Dimensional Heterojunction in a Nickel and Palladium Nanowire Complex,” Nature Communications 13 (2022): 1188, 10.1038/s41467-022-28875-8. PMC8897505 35246546

[24] K. Otsubo , Y. Wakabayashi , J. Ohara , et al., “Bottom‐Up Realization of a Porous Metal–Organic Nanotubular Assembly,” Nature Materials 10 (2011): 291–295, 10.1038/nmat2963. 21358649

[25] H. Moriyama , K. Otsubo , K. Sugimoto , S. Kawaguchi , and H. Kitagawa , “Observation of an Alternate Charge‐Polarization State in a One‐Dimensional Pt−Pt−I Chain Compound With a Bulky Pendant Ligand,” Angewandte Chemie International Edition 134 (2022): e202214108, 10.1002/ange.202214108. 36305378

[26] M. Mitsumi , T. Murase , H. Kishida , et al., “Metallic Behavior and Periodical Valence Ordering in a MMX Chain Compound, Pt_2_(EtCS_2_)_4_I,” Journal of the American Chemical Society 123 (2001): 11179–11192, 10.1021/ja010900v. 11697961

[27] N. Kimizuka , “Towards Self‐Assembling Inorganic Molecular Wires,” Advanced Materials 12 (2000): 1461–1463, 10.1002/1521-4095(200010)12:19<1461::AID-ADMA1461>3.0.CO;2-X.

[28] M. R. Mian , H. Iguchi , S. Takaishi , et al., “Multiple‐Hydrogen‐Bond Approach to Uncommon Pd(III) Oxidation State: A Pd–Br Chain With High Conductivity and Thermal Stability,” Journal of the American Chemical Society 139 (2017): 6562–6565, 10.1021/jacs.7b02558. 28467845

[29] Y. Wakabayashi , N. Wakabayashi , M. Yamashita , T. Manabe , and N. Matsushita , “Spatial Correlations in the Valence of Metal Ions in Ni_1‐x_Pd_x_(chxn)_2_Br_3_ ,” Journal of the Physical Society of Japan 68 (1999): 3948–3952, 10.1143/JPSJ.68.3948.

[30] M. R. Mian , M. Wakizaka , T. Yoshida , H. Iguchi , S. Takaishi , and M. Yamashita , “Recent Progress on Unusual Halogen‐Bridged Pd(III) Chain Compounds Realized by Weak Ligand Field and Triple Hydrogen Bond Approaches,” Coordination Chemistry Reviews 475 (2023): 214878, 10.1016/j.ccr.2022.214878.

[31] H. Kishida , H. Matsuzaki , H. Okamoto , et al., “Gigantic Optical Nonlinearity in One‐Dimensional Mott–Hubbard Insulators,” Nature 405 (2000): 929–932, 10.1038/35016036. 10879529

[32] H. Matsuzaki , M. Iwata , T. Miyamoto , et al., “Excitation‐Photon‐Energy Selectivity of Photoconversions in Halogen‐Bridged Pd‐Chain Compounds: Mott Insulator to Metal or Charge‐Density‐Wave State,” Physical Review Letters 113 (2014): 096403, 10.1103/PhysRevLett.113.096403. 25215998

[33] M. Yamashita , “Next Generation Multifunctional Nano‐Science of Advanced Metal Complexes With Quantum Effect and Nonlinearity,” Bulletin of the Chemical Society of Japan 94 (2021): 209–264, 10.1246/bcsj.20200257.

[34] H. Liang , K. Otsubo , and H. Kitagawa , “Dimensionally Extending From 1D MX–Chain to Ladder and Nanotube Systems: Structural and Electronic Properties,” Chemistry—A European Journal 30 (2024): e202402583, 10.1002/chem.202402583. 39276344

[35] S. Iwai , M. Ono , A. Maeda , et al., “Ultrafast Optical Switching to a Metallic State by Photoinduced Mott Transition in a Halogen‐Bridged Nickel‐Chain Compound,” Physical Review Letters 91 (2003): 057401, 10.1103/PhysRevLett.91.057401. 12906630

[36] Y. Wang , T. Zhang , R. Huang , et al., “Ultrasmall Carbon Nanotube‐Like Network in ZSM‐5 by Optimized Methane CVD,” Materials & Design 268 (2026): 116538, 10.1016/j.matdes.2026.116538.

[37] B. Zhang , T. Zhang , J. Pan , et al., “Peierls‐Type Metal‐Insulator Transition in Carbon Nanostructures,” Carbon 172 (2021): 106–111, 10.1016/j.carbon.2020.10.037.

[38] C. H. Wong , Z. Guo , K. C. Lam , et al., “Tuning Charge Density Waves and Magnetic Switching in Carbon Nanowires Encased in Boron Nitride Nanotubes,” Materials Horizons 12 (2025): 8205–8215, 10.1039/d5mh00664c. 40671604

[39] R. J. H. Clark , M. Kurmoo , H. J. Keller , B. Keppler , and U. Traeger , “Electronic and Resonance‐Raman Spectra of the Linear‐Chain Mixed‐Valence Platinum Complexes {[Pt(en)_2_][Pt(en)_2_X_2_]}_3_ [CuX_4_]_4_ [X = Cl (1) or Br (2)] and [Pt(pn)_2_][Pt(pn)Br_2_][Cu_3_Br_5_]_2_ (3) (en = ethane‐1,2‐diamine and pn = propane‐1,2‐diamine),” Journal of the Chemical Society, Dalton Transactions 9 (1980): 2498, 10.1039/DT9800002498.

[40] M. Yamashita and H. Okamoto , Material Designs and New Physical Properties in MX‐ and MMX‐Chain Compounds (Springer Science & Business Media, 2012).

[41] Y. Hamaue , R. Aoki , M. Yamashita , and S. Kida , “Electric Conduction Studies on Mixed‐Valence Complexes of Platinum and Palladium,” Inorganica Chimica Acta 54 (1981): L13–L14, 10.1016/S0020-1693(00)95369-6.

[42] M. Wakizaka , H. Tanaka , S. Takaishi , and M. Yamashita , “Superatomic Stabilization of Dinuclear Platinum (III) Through Iodide‐Bridged Five‐Center Ten‐Electron Bonding,” Inorganic Chemistry 64 (2024): 97–104, 10.1021/acs.inorgchem.4c04178. 39700064

[43] K. Prassides and P. N. Schatz , “Optical Properties of the Prototype II–IV Mixed‐valence System Wolffram's Red Salt: A One Mode Treatment,” Chemical Physics Letters 178 (1991): 227–234, 10.1016/0009-2614(91)87061-F.

[44] K. Prassides , P. N. Schatz , K. Y. Wong , and P. Day , “Vibronic Coupling Model for Mixed‐Valence Compounds. Extension to Two‐Site Two‐Electron Systems,” Journal of Physical Chemistry 90 (1986): 5588–5597, 10.1021/j100280a023.

[45] K. Nasu , “Extended Peierls‐Hubbard Model for One‐Dimensional N‐Sites N‐Electrons System. I. Phase Diagram by Mean Field Theory,” Journal of the Physical Society of Japan 52 (1983): 3865–3873, 10.1143/JPSJ.52.3865.

[46] K. Uemura , N. Uesugi , A. Matsuyama , M. Ebihara , H. Yoshikawa , and K. Awaga , “Integration of Paramagnetic Diruthenium Complexes Into an Extended Chain by Heterometallic Metal–Metal Bonds With Diplatinum Complexes,” Inorganic Chemistry 55 (2016): 7003–7011, 10.1021/acs.inorgchem.6b00741. 27348516

[47] P. Papadia , K. Micoli , A. Barbanente , et al., “Platinum (IV) Complexes of Trans‐1,2‐Diamino‐4‐Cyclohexene: Prodrugs Affording an Oxaliplatin Analogue That Overcomes Cancer Resistance,” International Journal of Molecular Sciences 21 (2020): 2325, 10.3390/ijms21072325. PMC7177638 32230896

[48] Y. Luo , N. Zhou , Y. Zhang , et al., “Tailoring the Electron Pairing Process in a Pt–I Charge‐Density‐Wave Chain,” Journal of the American Chemical Society (2026): 30466–30475, 10.1021/jacs.6c09941. PMC13397895 42420162

