# Supplementary material for: Suppression of Peierls Instability in a Metal−Halide Porous Framework
Source: Angew Chem Int Ed Engl. 2026 Aug 23;65(42):e7724982. doi: 10.1002/anie.7724982 (PMC13653625; doi:10.1002/anie.7724982)
Supplement: Supplementary file 1 [file ANIE-65-e7724982-s001.pdf]

Supporting Information
©Wiley-VCH 2021
69451 Weinheim, Germany

Suppression of Peierls Instability in a Metal−Halide Porous Framework

Ning Zhou, Yangbo Zhang, Ying-Fan Tan and Qingyun Wan*

Dr. Ning Zhou, Mr. Yangbo Zhang, Mr. Ying-Fan Tan, and Prof, Dr. Qingyun Wan

Department of Chemistry,

The Chinese University of Hong Kong

Hong Kong SAR 999077, China

E-mail: qingyunwan@cuhk.edu.hk

Experimental Procedures

Methods and Measurements: All starting materials were purchased from commercial sources and used as received. The solvents used for synthesis were of analytical grade unless stated otherwise. $Pt(en)_2Cl_2$ was synthesized following the procedure previously reported in the literature [39]. X-ray crystal data were collected on a Bruker SMART CCD diffractometer and a Bruker KAPPA APEX II diffractometer with graphite-monochromatized Mo-K_α radiation (λ = 0.71073 Å) at 100 K. An empirical absorption correction was applied using the SADABS program. The structures were solved by direct phase determination using the computer program SHELX-97 and refined by all-matrix least-squares with anisotropic thermal parameters for the non-hydrogen atoms. Hydrogen atoms were introduced in their idealized positions and included in structure factor calculations with assigned isotropic temperature factors. The X-ray powder diffraction (XPRD) pattern of crystal was simulated using Mercury software. XPRD pattern of crystal powder was recorded using Rigaku MiniFlex600. Thermogravimetric analysis (TGA) curve was performed on a Heson HS-TGA-101 analyzer. The elemental analysis (EA) results were obtained using a Thermo FLASH2000 elemental analyzer. Absorption spectrum was measured on crystal powder samples using a Lambda 750s UV-vis-NIR spectrometer. Raman spectra were measured using a Horiba Scientific XploRA Plus spectrometer. X-ray photoelectron spectroscopy (XPS) spectra were acquired on a Thermo Scientific K-Alpha spectrometer. Temperature dependence χT was measured on Quantum Design Magnetic Properties Measurement System (MPMS). Two-probe electrical measurements were performed with a Keithley 4200-SCS parameter analyzer. Four-probe electrical measurement was performed with Physical Property Measurement System Quantum Design PPMS.

Synthesis of Compound 1: $\{[Pt(en)_2][PtBr_2(en)_2]\}_3[CuBr_4]_4$. $Pt(en)_2Cl_2$ (10 mg) and CuBr (15 mg) were dissolved independently in 5 mL of a 500 mg/mL KBr aqueous solution under moderate heating. Upon cooling to ambient temperature, both solutions were filtered and partitioned equally into five separate vials. The CuBr solution was then added dropwise to the $Pt(en)_2Cl_2$ solution. After a two-week induction period, copper-green acicular crystals with a distinctive metallic luster precipitated. The resulting product was isolated via filtration and rinsed sequentially with deionized water and acetone, yielding the final crystalline material (12 mg, 72% yield). These crystals were subsequently collected for structural characterization and physicochemical analysis. Elemental analysis (EA): Compound **1**·$12H_2O$: Anal. Calcd for $C_{24}H_{120}Br_{22}Cu_4N_{24}Pt_6O_{12}$: C, 6.99; H, 2.82; N, 8.16; Found: C, 6.68; H, 2.85; N, 8.07.

Synthesis of Compound 2: $[PtI(en)_2]_3(CuI_4)_2CuI$. Compound **2** was synthesized following a procedure analogous to that described for Compound **1**, with the substitution of CuI and KI. After two weeks, elongated, brown crystals exhibiting a characteristic metallic luster precipitated within the vessel. The product was isolated through filtration, followed by washing with deionized water and acetone, respectively, to yield the final crystals (14 mg, 60% yield). The resulting crystals were collected and reserved for subsequent structural characterization and physicochemical property test. Elemental analysis (EA): Compound **2**·$5O_2$: Anal. Calcd for $C_{12}H_{48}Cu_3I_{12}N_{12}Pt_3O_{10}$: C, 4.59; H, 1.53; N, 5.35; Found: C, 4.45; H, 1.48; N, 5.40.

Computational Details

The structures of Pt_4I_5 and Pt_4I_5-Cu_4I_{12} of compound **2** used for Density Functional Theory (DFT) and Time-Dependent Density Functional Theory (TDDFT) calculations were taken directly from X-ray crystal structures without geometry optimization. All DFT calculations were performed with Gaussian 16, Revision C.02 [49]. The PBE0 functional [50] together with Grimme's D3BJ dispersion correction [51,52] was employed for all calculations. The 6-31G(d) basis set [53,54] was used for light elements (H, C, N, Cu), while the Stuttgart/Dresden (SDD) relativistic effective core potential and its associated valence basis set [55,56] were applied for heavy elements (Pt, I) to account for scalar relativistic effects. Molecular orbital diagrams were plotted using Multiwfn 3.8 [57] and VMD 1.9.3 [58]. Electrostatic potential maps were produced with GaussView 6.1 [59].

Supplementary Figures and Data

Table S1 Crystal data and structure refinements for Compound **1** (CCDC: 2545144).

	**$\{[Pt(en)_2][Pt(en)_2Br_2]\}_3[CuBr_4]_4$**
Empirical formula	$C_{24}H_{96}Br_{22}Cu_4N_{24}Pt_6$
Formula weight	3903.96
Temperature / K	293(2)
Crystal system	Trigonal
Space group	P3
a / Å	16.018(3)
b / Å	16.018(3)
c / Å	10.941(2)
α / °	90.00
β / °	90.00
γ / °	120.00
Volume / $Å^3$	2431.1(10)
Z	1
ρ_{calc} g/cm^3	2.667
μ/mm^{-1}	18.523
F(000)	1762
Crystal size / mm^3	0.400×0.300×0.200
Radiation	MoKα (λ = 0.71073)
2θ range for data collection/°	2.371 to 28.227
Index ranges	−21 ≤ h ≤ 21, −21 ≤ k ≤ 21, −14 ≤ l ≤ 13
Reflections collected	48194
Independent reflections	7486 [R_{int} = 0.0563]
Data/restraints/parameters	7486/1/241
Goodness-of-fit on F^2	1.135
Final R indexes [R>=2σ(I)]	R1 = 0.0597, wR2 = 0.1754
Final R indexes [all data]	R1 = 0.0642, wR2 = 0.1813
Largest diff. peak/hole / $eÅ^{-3}$	22.960/−4.0001

Table S2 Crystal data and structure refinements for Compound **2** (CCDC: 2545151).

	**$CuI[Pt(en)_2I]_3(CuI_4)_2CuI$**
Empirical formula	$C_{12}H_{48}Cu_3I_{12}N_{12}Pt_3$
Formula weight	2659.31
Temperature / K	293(2)
Crystal system	Hexagonal
Space group	P63/mmc
a / Å	16.696(3)
b / Å	16.696(3)
c / Å	11.534(2)
α / °	90.00
β / °	90.00
γ / °	120.00
Volume / $Å^3$	2784.5(12)
Z	2
ρ_{calc} g/cm^3	3.172
μ/mm^{-1}	15.306
F(000)	2322
Crystal size / mm^3	0.400×0.300×0.200
Radiation	MoKα (λ = 0.71073)
2θ range for data collection/°	2.259 to 28.295
Index ranges	−22 ≤ h ≤ 22, −22 ≤ k ≤ 22, −14 ≤ l ≤ 15
Reflections collected	72292
Independent reflections	1279 [R_{int} = 0.0457]
Data/restraints/parameters	1279/0/56
Goodness-of-fit on F^2	1.095
Final R indexes [R>=2σ(I)]	R1 = 0.0528, wR2 = 0.1727
Final R indexes [all data]	R1 = 0.0542, wR2 = 0.1750
Largest diff. peak/hole / $eÅ^{-3}$	3.473/−6.202

Table S3 Crystal data and structure refinements for Compound **2** at 100 K (CCDC: 2580141). (Note: a strong positional disorder was evident in the Cu–I chain, for which the Cu site occupancy was fixed at 1.67 throughout the X-ray structure refinement.)

	**$CuI[Pt(en)_2I]_3(CuI_4)_2CuI$**
Empirical formula	$C_4 H_{16} Cu_{1.67} I_4 N_4 Pt$
Formula weight	928.80
Temperature	100(2) K
Wavelength	0.71073 Å
Crystal system	Trigonal
Space group	P-3
Unit cell dimensions	a = 16.5861(13) Å
	b = 16.5861(13) Å
	c = 5.7266(6) Å
Volume	1364.3(3) $Å^3$
Z	3
Density (calculated)	3.391 Mg/m^3
Absorption coefficient	16.379 mm^{-1}
F(000)	1219
Crystal size	0.400 x 0.300 x 0.200 mm^3
Theta range for data collection	4.916 to 27.918°.
Index ranges	-21<=h<=21, -21<=k<=21, -7<=l<=7
Reflections collected	25032
Independent reflections	2151 [R(int) = 0.0934]
Completeness to theta = 25.242°	97.4 %
Absorption correction	multi-scan
Max. and min. transmission	0.7456 and 0.4534
Refinement method	Full-matrix least-squares on F^2
Data / restraints / parameters	2151 / 1 / 102
Goodness-of-fit on F^2	1.060
Final R indices [I>2sigma(I)]	R1 = 0.0509, wR2 = 0.1301
R indices (all data)	R1 = 0.0581, wR2 = 0.1368
Largest diff. peak and hole	3.511 and -4.412 e.Å-3

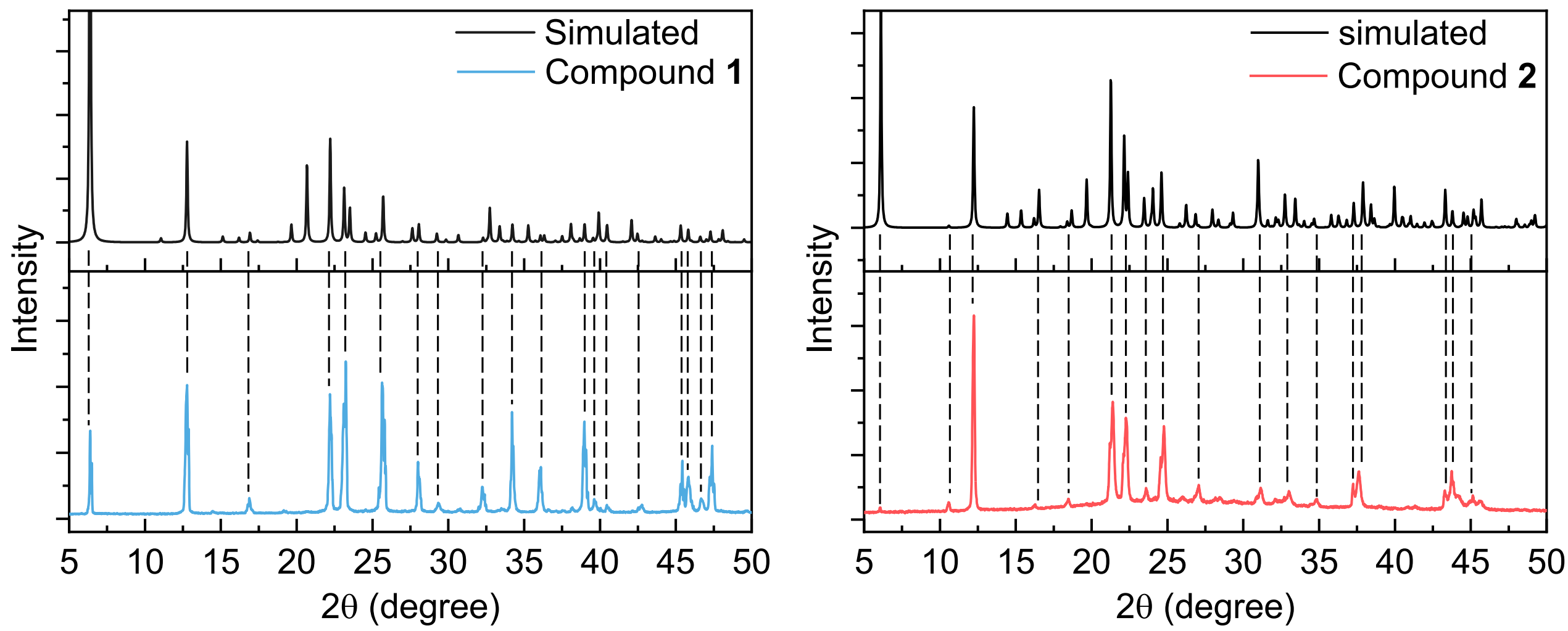

Figure S1 Comparison of experimental (bottom) and simulated (top) PXPRD patterns for compound **1** and compound **2**.

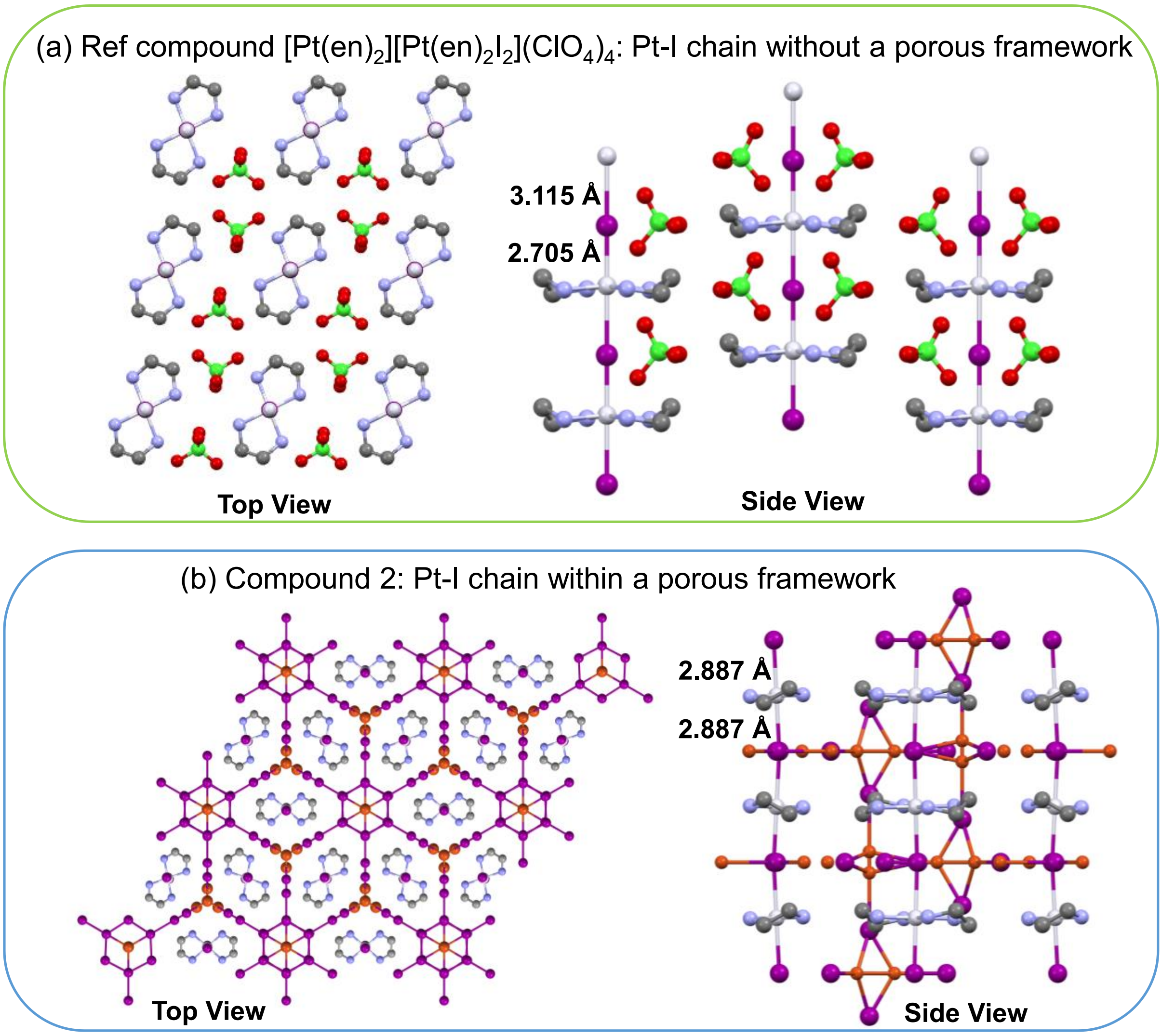

Figure S2 X-ray crystal structure comparison between (a) reference compound $[Pt(en)_2][Pt(en)_2I_2](ClO_4)_4$ and (b) compound **2**. [40,41]

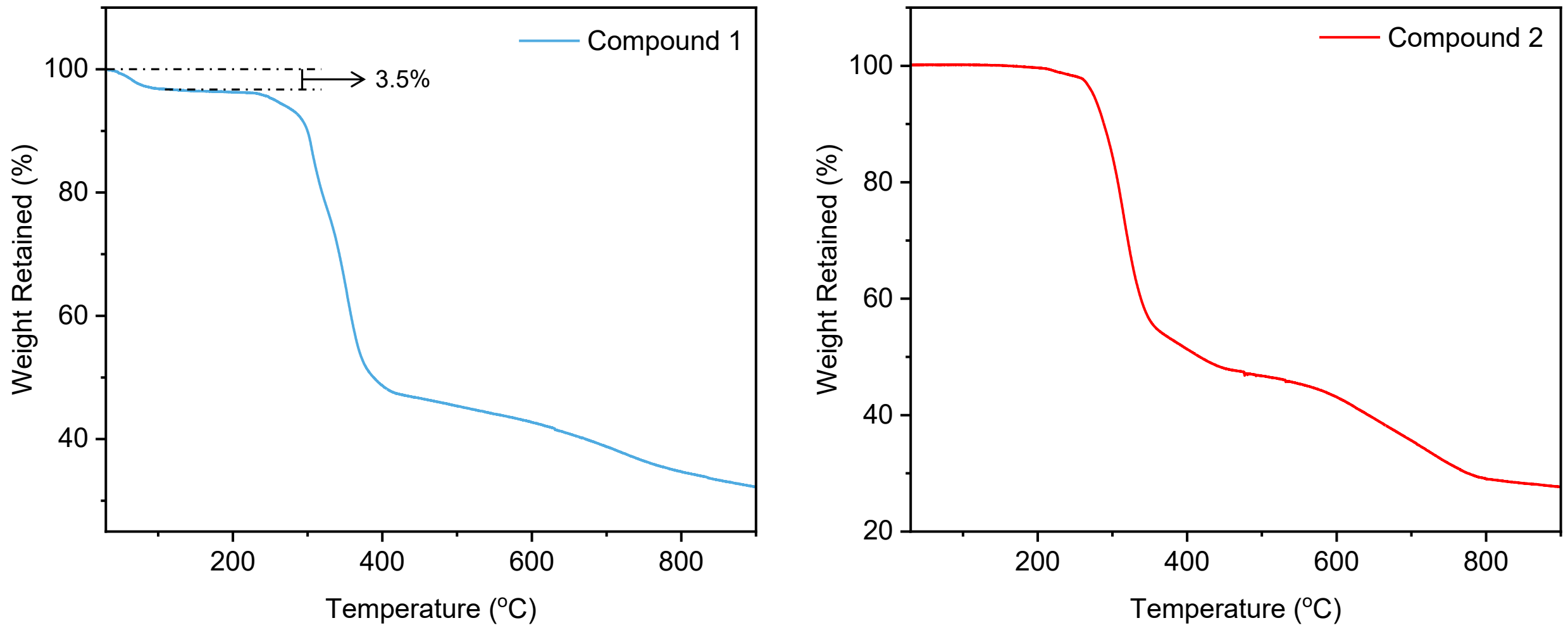

Figure S3 Thermogravimetric Analysis (TGA) results. TGA of compound **1** and compound **2**.

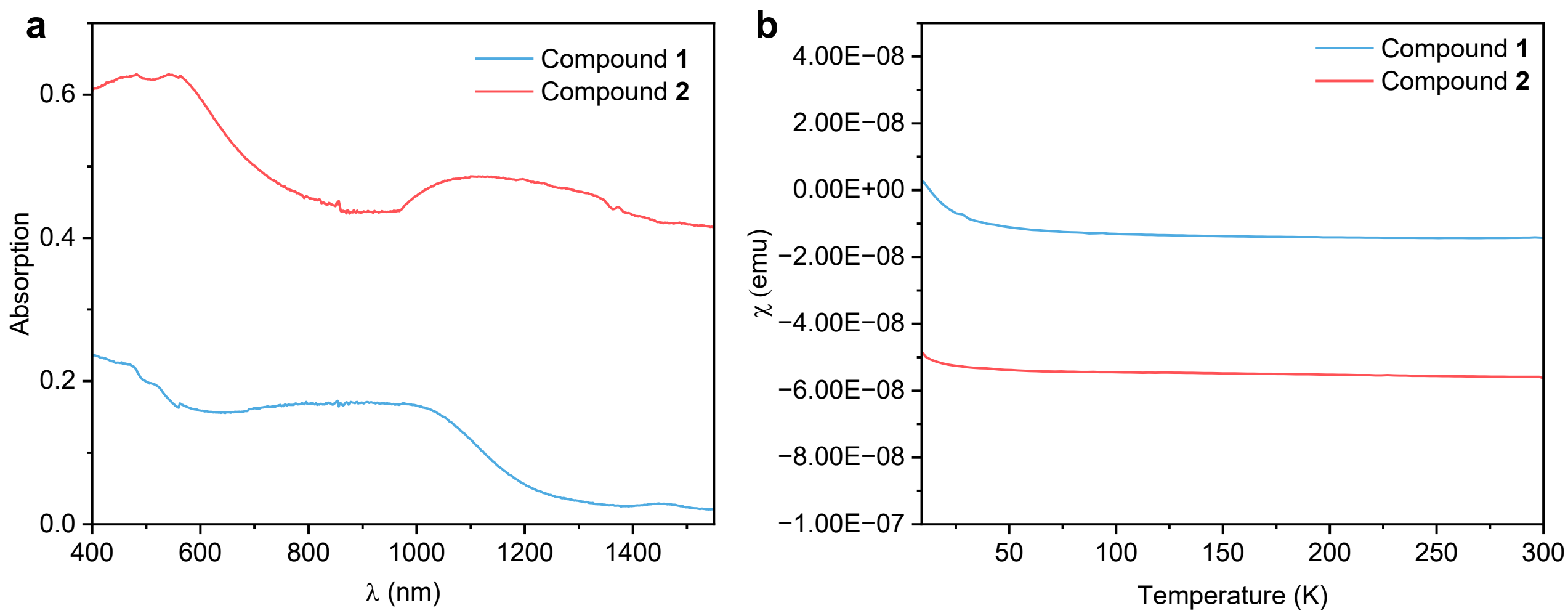

Figure S4 (a) Absorption spectra of the crystals powder sample for compound **1** and **2** at room temperature. (b) Temperature-dependent magnetic susceptibility (χ) of compound **1** and **2** under an applied field of 1000 Oe.

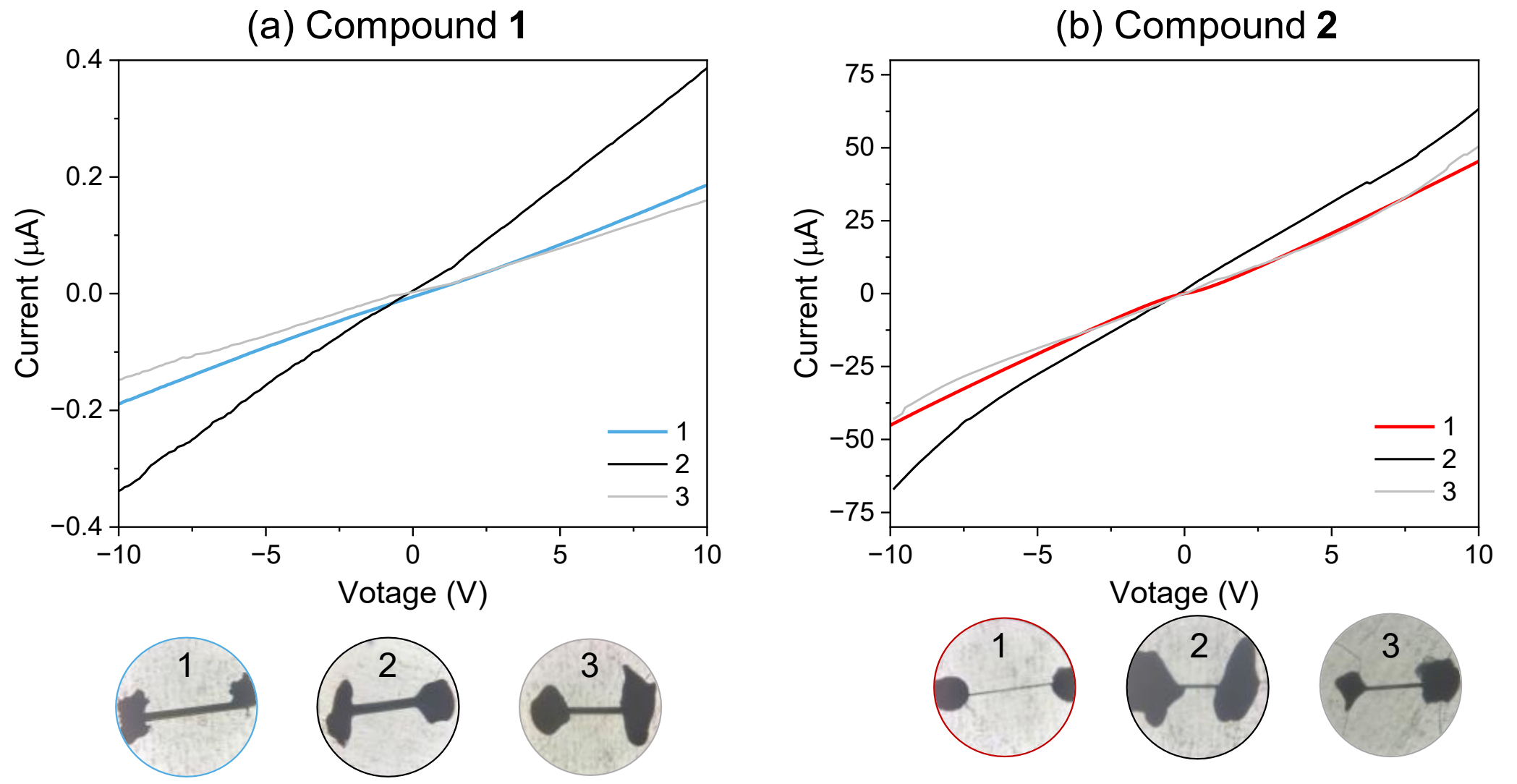

Number of tests	σ (Ω^{-1} cm^{-1})
1	4.83×10^{-6}
2	2.29×10^{-6}
3	3.78×10^{-6}

Number of tests	σ (Ω^{-1} cm^{-1})
1	4.25×10^{-2}
2	1.13×10^{-2}
3	1.38×10^{-2}

Figure S5. Two-probe I–V curves measured at room temperature on three single-crystal samples, with the device structure illustrated in the inset.

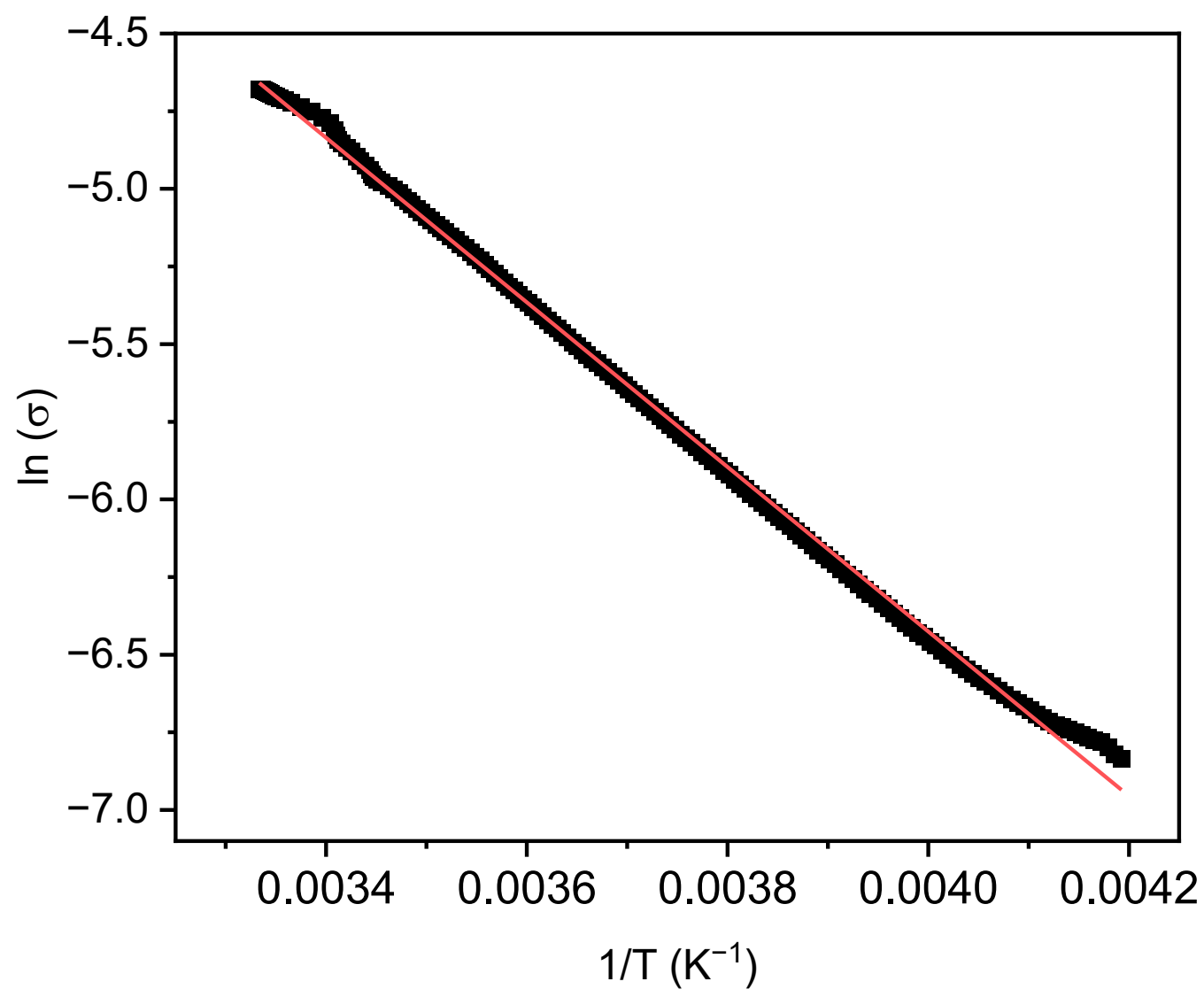

Figure S6. Plot of ln(σ) versus reciprocal temperature, along with its linear fit (red line).

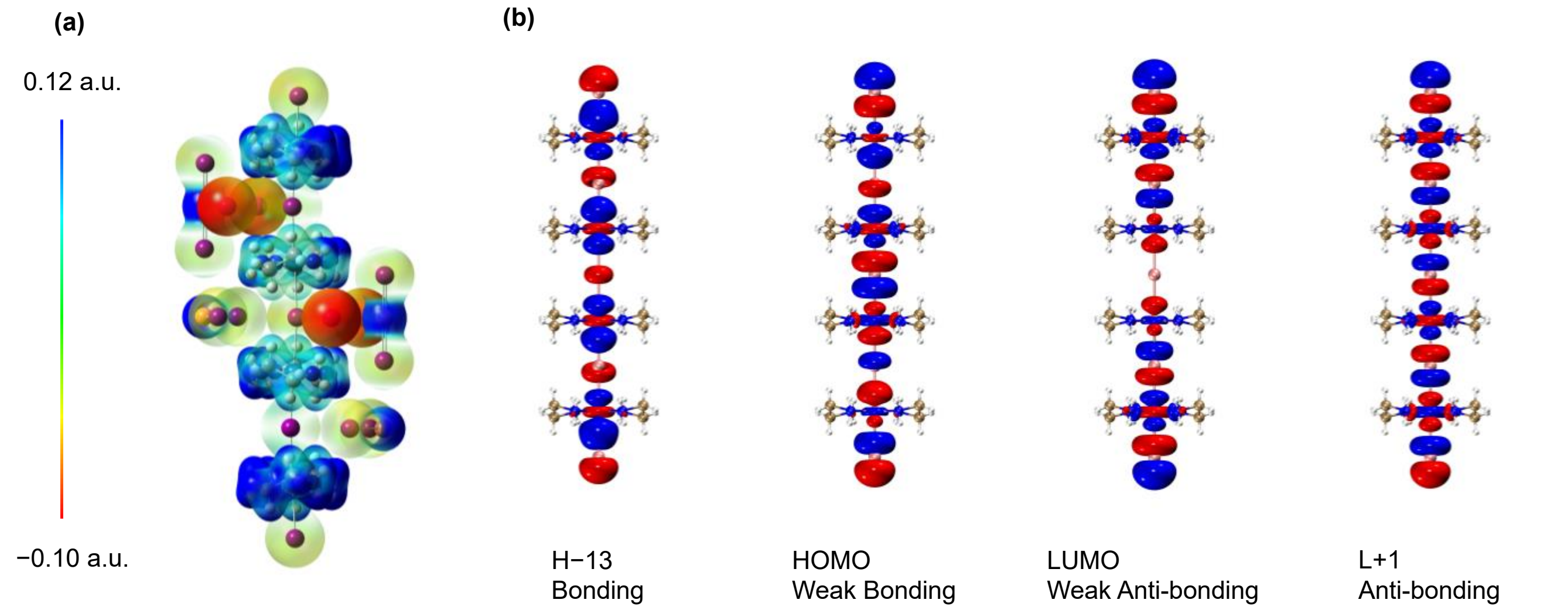

Figure S7. (a) Calculated ESP (electrostatic potential) maps of compound **2**. Red region: negative charge and blue region: positive charge. (b) Calculated MO (molecular orbital) to show the Pt−Pt bonding, weakly bonding, weakly antibonding, and antibonding interaction for the compound **2** with the chain length of Pt_4I_5.

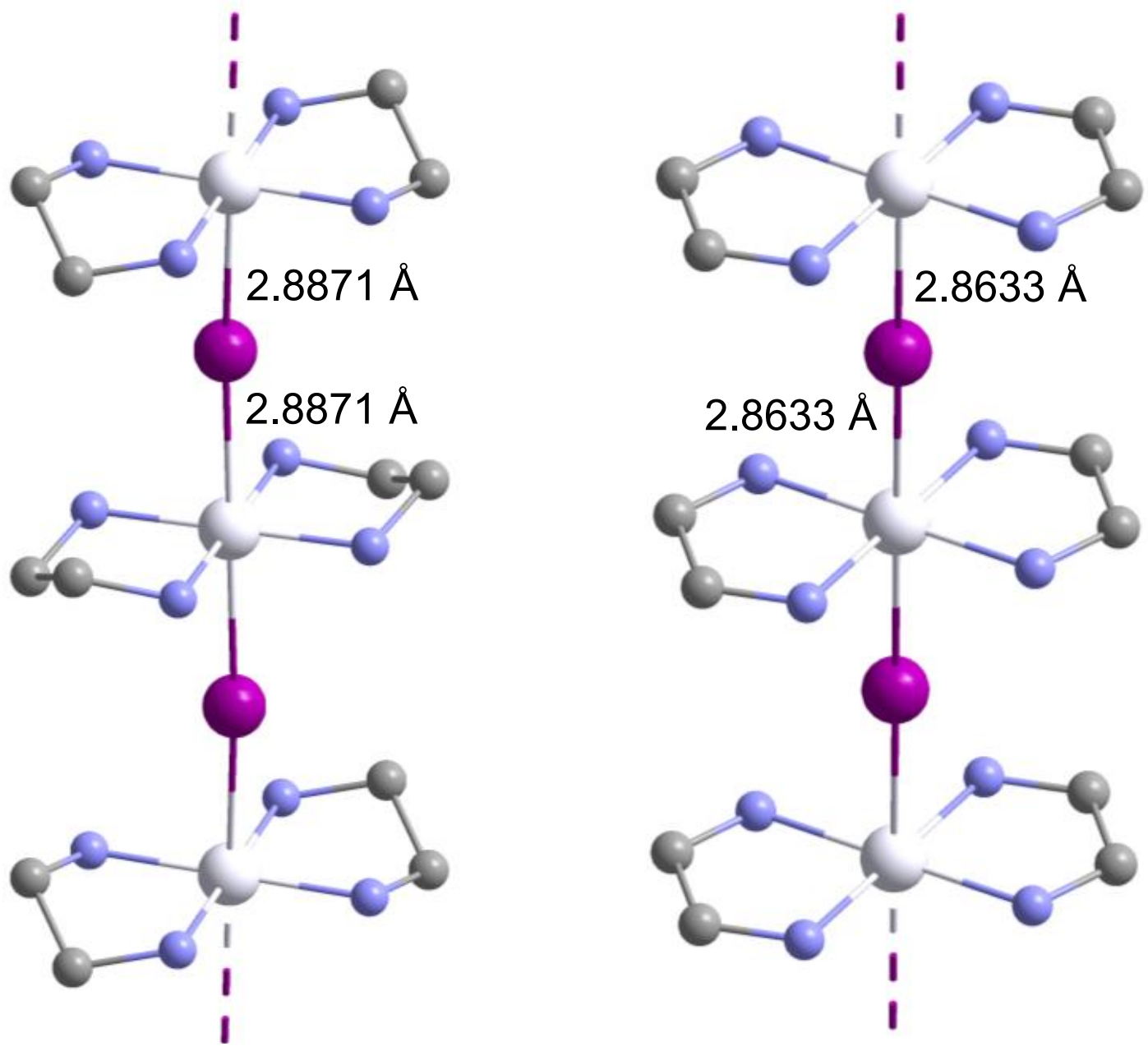

Figure S8. X-ray crystal structure of compound **2** at 293 K (left) and 100 K (right).

[39] R. J. H. Clark, M. Kurmoo, H. J. Keller, B. Keppler, and U. Traeger, “Electronic and Resonance-Raman Spectra of the Linear-Chain Mixed-Valence Platinum Complexes {$[Pt(en)_2][Pt(en)_2X_2]\}_3$ $[CuX_4]_4$ [X = Cl (1) or Br (2)] and $[Pt(pn)_2][Pt(pn)Br_2][Cu_3Br_5]_2$ (3) (en=ethane-1,2-diamine and pn=propane-1,2-diamine),” *Journal of the Chemical Society, Dalton Transactions* (1980): 2498−2502, https://doi.org/10.1039/DT9800002498.

[40] M. Yamashita and H. Okamoto, Material Designs and New Physical Properties in MX- and MMX-Chain Compounds, Springer Science & Business Media (2012).

[41] Y. Hamaue, R. Aoki, M. Yamashita, et al., “Electric Conduction Studies on Mixed-Valence Complexes of Platinum and Palladium,” *Inorganica Chimica Acta* 54 (1981): L13−L14, https://doi.org/10.1016/S0020-1693(00)95369-6.

[49] M. J. Frisch, G. W. Trucks, H. B. Schlegel, et al., “Gaussian 16,” Gaussian, Inc., Wallingford CT (2016).

[50] C. Adamo and V. Barone, “Toward Reliable Density Functional Methods without Adjustable Parameters: The PBE0 Model,” *The Journal of Chemical Physics* 110 (1999): 6158−6170, https://doi.org/10.1063/1.478522.

[51] S. Grimme, J. Antony, S. Ehrlich, et al., “A Consistent and Accurate Ab Initio Parametrization of Density Functional Dispersion Correction (DFT-D) for the 94 Elements H−Pu,” *The Journal of Chemical Physics* 132 (2010): 154104, https://doi.org/10.1063/1.3382344.

[52] S. Grimme, S. Ehrlich and L. Goerigk, “Effect of the Damping Function in Dispersion Corrected Density Functional Theory,” *Journal of Computational Chemistry* 32 (2011): 1456−1465, https://doi.org/10.1002/jcc.21759.

[53] W. J. Hehre, R. Ditchfield, J. A. Pople, “Self-Consistent Molecular Orbital Methods. XII. Further Extensions of Gaussian-Type Basis Sets for Use in Molecular Orbital Studies of Organic Molecules,” *The Journal of Chemical Physics* 56 (1972): 2257−2261, https://doi.org/10.1063/1.1677527.

[54] P. C. Hariharan and J. A. Pople, “The Influence of Polarization Functions on Molecular Orbital Hydrogenation Energies,” *Theoretica Chimica Acta* 28 (1973): 213−222, https://doi.org/10.1007/BF00533485.

[55] G. Igel-Mann, H. Stoll, H. Preuss, “Pseudopotentials for Main Group Elements (IIIa Through VIIa),” *Molecular Physics* 65 (1988): 1321−1328, https://doi.org/10.1080/00268978800101811.

[56] D. Andrae, U. Häußermann, M. Dolg, et al., “Energy-Adjusted Ab Initio Pseudopotentials for the Second and Third Row Transition Elements,” *Theoretica Chimica Acta* 77 (1990): 123−141, https://doi.org/10.1007/BF01114537.

[57] T. Lu, F. Chen, “Multiwfn: A Multifunctional Wavefunction Analyzer,” *Journal of Computational Chemistry* 33 (2012): 580−592, https://doi.org/10.1002/jcc.22885.

[58] W. Humphrey, A. Dalke, K. Schulten, “VMD: Visual Molecular Dynamics,” *Journal of Molecular Graphics* 14 (1996): 33−38, https://doi.org/10.1016/0263-7855(96)00018-5.

[59] R. Dennington, T. A. Keith, J. M. Millam, “GaussView, Version 6.1,” Semichem Inc., Shawnee Mission, KS (2016).
